# A closer look at the role of teacher emotional support, L2 grit, and burnout in foreign language achievement

**DOI:** 10.1186/s40359-026-04734-9

**Published:** 2026-05-09

**Authors:** Tingting Xie, Meihua Chen

**Affiliations:** School of Foreign Languages, Southeast University, Nanjing, China

**Keywords:** Teacher emotional support, L2 grit, Foreign language learning burnout, Foreign language achievement

## Abstract

**Background:**

Acquiring a foreign language (FL) is an enduring and challenging process which can be facilitated by a supportive learning environment, positive individual traits, and emotional well-being. To gain insights into how these factors shape FL learning, the study sought to explore the intricate interplay among environmental factors (i.e., teacher emotional support), personal traits (i.e., L2 grit), emotional well-being (i.e., foreign language learning burnout), and FL achievement.

**Method:**

We conducted a cross-sectional survey and recruited 1060 postgraduate EFL (English as a Foreign Language) learners using convenience sampling. Participants completed an online survey composed of scales measuring perceived teacher emotional support, L2 grit, and foreign language learning burnout. Their final course scores were used to assess FL achievement. Confirmatory factor analysis (CFA) was conducted to examine the construct validity of the measures. Structural equation modelling (SEM) was performed to test the hypothesized relationships among the variables.

**Results:**

SEM results indicated that both teacher emotional support and L2 grit significantly predicted FL achievement, but only indirectly through FL learning burnout. In comparison of their predictive effects, consistency of interest (one facet of L2 grit) showed the largest effect on FL burnout and achievement, followed by perseverance of effort (the other L2 grit facet), with teacher emotional support demonstrating the smallest effect. Moreover, the mediation analysis revealed that only two burnout components (i.e., emotional exhaustion and reduced efficacy) showed significant mediating effects, with reduced efficacy emerging as the stronger mediator, while cynicism was not a significant mediator.

**Conclusion:**

The findings highlight the importance of teacher emotional support and L2 grit, especially consistency of interest, in alleviating FL learning burnout and improving FL achievement. Based on these findings, suggestions and implications for future studies are presented.

## Introduction

The rise of positive psychology in second language acquisition (SLA) calls for a holistic language education approach that values both FL learners’ achievement and well-being [1]. FL learning is highly demanding and often psychologically unsettling, due to limited exposure to target language, obligatory and structured nature of learning activities, and pressure associated with language input and output. These demands, coupled with insufficient coping resources, leave FL learners susceptible to burnout, a key indicator of diminished well-being [1]. Typically defined as emotional exhaustion, cynicism, and reduced efficacy in the specific domain of FL learning [1], foreign language learning burnout has also been discovered to be closely associated with FL achievement [2]. To improve both EFL learners’ psychological well-being and FL achievement, it is necessary to investigate how to mitigate burnout in FL learning or help burned-out FL learners recover.

The antecedents of FL learning burnout, along with their impact on language learning outcomes, can be better understood within the demands-resources framework of burnout. Burnout in academic contexts, as the model suggests, emerges when individuals do not have sufficient environmental and personal resources to cope with demands and stressors in academic activities. The state of burnout, in turn, leads to poor academic performance. Conversely, the availability of environmental and personal resources, such as positive learning environment and individual traits, help alleviate students’ burnout, which further enhances positive academic outcomes. Teacher emotional support, represented as teachers’ care, respect, and empathy [3, 4], is examined as an environmental resource, given that FL learning primarily occurs in language classrooms, where teachers play a central role in shaping the learning experience. Emotionally supportive teachers often satisfy students’ emotional and psychological needs and sustain functioning, thereby protecting students from feeling burned out [5, 6] and improving academic achievement [7]. Among personal resources, L2 grit, the capacity to maintain consistent passion and endeavor in L2 learning despite setbacks [8], might be an effective buffer against burnout. Consistent passion keeps FL learning meaningful and buffers cynicism and energy depletion, while sustained effort supports self-regulation and progress that reduce inefficacy, thereby mitigating burnout [6]. Taken together, the current study explores how teacher emotional support and L2 grit, as key environmental and personal resources, buffer FL learners’ burnout, thereby indirectly enhancing FL achievement. Specifically, taking the demands-resources perspective, the study aims to investigate how teacher emotional support and L2 grit predict FL learning burnout and achievement, and then investigate whether burnout mediates their predictive effects on FL achievement. Moreover, to gain a closer insight into the roles of FL learning burnout and L2 grit, the study explores whether different dimensions of the two constructs exert different effects on FL achievement. The findings contribute to the field by identifying which dimensions of FL learning burnout and L2 grit are closely associated with one another and with FL learning outcomes, and therefore, may deserve greater attention in foreign language learning and teaching.

## Literature review

### The demands-resources model and foreign language learning burnout

The demands-resources model is a widely used framework for explaining the development of burnout, which was initially proposed in professional settings [9] and later extended to and validated in the fields of general education [10] and FL learning [6, 11, 12]. The core assumption of the model is that burnout is an outcome of the imbalance between demands (aspects of environment that require sustained effort and consume energy; e.g., heavy workload) and resources (environmental and personal factors that help individuals meet demands, attain goals, and achieve personal growth; e.g., performance feedback and self-efficacy). Another assumption is that demands and resources trigger two distinct processes: a health impairment process and a motivational process. The former refers to the process where chronic high demands drain individuals’ mental and physical resources, causing strain and exhaustion, thereby increasing burnout risks. The process further posits that when students are burned out, they lack psychological resources to invest effort which undermines functioning and performance and ultimately leads to negative outcomes (e.g., poor academic achievement). In contrast, the motivational process suggests that sufficient resources buffer the negative impacts of demands by protecting individuals’ energy and fostering sustained engagement in learning, leading to lower levels of burnout and positive performance. This dual-process framework aligns with the growing research at the intersection of positive psychology (PP) and SLA, which has increasingly attended to both the facilitative role of positive psychological resources (e.g., grit, enjoyment) and the debilitating effects of negative experiences (e.g., anxiety, burnout) in language learning [13]. More recently, the PP 2.0 perspective has called for examining these positive and negative dimensions simultaneously rather than in isolation, arguing that learner flourishing emerges from the dynamic interplay between the two [14]. The demands-resources model adopted in the present study resonates with this integrative view, as it accounts for both the resource-building motivational process and the energy-depleting burnout process within a single framework.

The model provides a useful perspective for understanding student burnout in EFL contexts. In FL learning, learners face language learning demands including limited exposure to the target language, sustained pressure to process input and output, and frequent evaluations. When such demands exceed their coping resources, FL learners might feel emotionally exhausted, detached, and incompetent in FL learning (i.e., burned out). In response to recent calls to investigate individual differences in language-specific domain [8], and with further support from meta-analytic evidence confirming the detrimental correlates of burnout across FL educational contexts [15], foreign language learning burnout has attracted increasing attention from SLA scholars. Foreign language learning burnout is defined as an adverse psychological state characterized by emotional exhaustion, cynicism, and reduced efficacy in FL learning [1]. Emotional exhaustion is defined as feelings of being overwhelmed or mentally drained due to the demands of FL learning or classes; cynicism involves developing cynical and detached attitudes towards language learning; reduced efficacy denotes a sense of incompetence, low self-esteem, and a diminished sense of accomplishment in FL learning [1, 16]. From a demands-resources perspective, this undesirable psychological state can be alleviated when FL learners have sufficient resources, both personal and environmental, to cope with the demands and stressors inherent in FL learning. In SLA, personal resources, including trait emotional intelligence [17], mindfulness [18], and L2 grit [6], have been identified as protective factors that help students withstand stressors and reduce burnout in FL learning. At the environmental level, prior research suggested that resources such as positive teacher-student relationship [2], teacher and peer support [19], as well as teacher immediacy and stroke behavior [20] served as effective buffers against FL learning burnout. Additionally, FL learning burnout has been found to correlate with various undesirable psychological outcomes, including reduced engagement, motivation, and willingness to communicate in FL learning [21, 22]. More importantly, a few studies revealed its correlation with diminished FL achievement [2, 17]. These findings were consistent with the energy-depleting process suggesting that burnout undermines psychological resources needed for effective performance.

Taken together, the demands-resources model and empirical findings support examining the relationship between FL learning burnout, teacher emotional support and L2 grit (as antecedents of FL learning burnout), and FL achievement (as academic outcomes of FL learning burnout) within an integrated framework. Yet, no studies, to the best of our knowledge, have examined them together in one single model, neglecting the intricate interplay among them. Additionally, most studies mentioned above focused on secondary school or undergraduate EFL learners, suffering from biased sampling. Postgraduate EAP learners, shifting from the general English course focused on their English proficiency to EAP courses centered on academic genres and research-based writing in which they have little prior training, often face substantial linguistic and discourse-related challenges (e.g., academic style, cohesion, and academic community practices) as well as broader adjustment demands such as meeting new expectations and seeking social support in a new academic environment [23, 24]. Challenged by these distinctive demands, they may exhibit unique burnout profiles and patterns of association with key resources and outcomes. In response to the call to diversify participant samples in applied linguistics [25, 26], more attention should be paid to this important yet underexplored learner population.

### Perceived teacher emotional support: environmental resource in the demands-resources model

Teacher emotional support is defined as the genuine care, respect, and empathy demonstrated by teachers towards their students, fostering warm, trusting, and affective relationships that satisfy students’ emotional needs [3, 4]. Teacher emotional support has been discovered to make students feel more connected and engaged in academic learning, satisfying their psychological needs and supporting adaptive learning [27]. Recent research supported the positive role of teachers’ emotional presence in EFL contexts. Teachers’ affective scaffolding and teacher enthusiasm, constructs that share conceptual ground with teacher emotional support, have been linked to favourable psycho-affective outcomes, including stronger engagement, psychological well-being, and self-efficacy [28, 29]. More directly relevant to the present study, research on EFL teacher emotional support has evidenced its significant role in promoting positive emotional and psychological outcomes in L2 learning, including L2 willingness to communicate, engagement, and motivation [30–33]. From a demands-resources perspective, teacher emotional support, by reducing students’ alienation and disengagement, can be viewed as a key classroom-level resource that potentially buffers the strain of high demands and reduces burnout [34]. Empirical studies in EFL settings provided evidence for the protective role of teacher emotional support in buffering FL learning burnout. Wu et al. [6] and Karimi and Fallah [11] identified both a direct impact of teacher emotional support on EFL learners’ burnout and an indirect impact mediated by constructs like motivation and grit. He et al. [35] also reported a negative association between teacher emotional support and EFL learners’ burnout in online learning contexts.

Existing studies have underscored the advantageous role of teacher emotional support in foreign language learning, especially in enhancing learners’ emotional and psychological outcomes such as engagement, emotions, and motivation [27, 31]. Yet, only a few studies connected teacher emotional support to FL achievement, a crucial yardstick of FL learning outcomes, and the association remains unclear due to mixed findings. Ghaith [4] revealed that teacher emotional support failed to influence FL achievement. Li et al. [36] incorporated academic buoyancy into their analysis and reported that teacher emotional support did not directly impact FL achievement but had an indirect effect through academic buoyancy. In contrast, Ma et al. [2] demonstrated that supportive teacher-student relationships not only had an indirect positive impact on FL achievement through burnout and enjoyment but also directly enhanced it. These inconsistent findings may be attributed to different contexts and participants, as well as the incorporation of varying constructs. This highlights the need for further empirical research into how teacher emotional support influences language learning outcomes across diverse learner populations and contexts, as well as the significance of investigating additional key constructs (e.g., FL learning burnout) from mainstream EFL studies that may influence this relationship. Hence, this study investigates how teacher emotional support relates to FL achievement and whether FL burnout mediates this relationship.

### L2 Grit: personal resource in the demands-resources model

L2 grit is conceptualized as perseverance of effort (POE) and consistency of interest (COI) in L2 learning. POE refers to the tendency to sustain efforts over an extended period in L2 learning despite setbacks, while COI denotes the continued enthusiasm and interest for L2 learning over time [8]. As a personal resource suggested by the demands-resources model, L2 grit has been investigated as a protective factor mitigating EFL learners’ burnout. For instance, Fan et al. [37] and Hu et al. [38] explored the possible mediating role of L2 grit in the relationship between language mindset and burnout, and indicated that L2 grit significantly and negatively predicted EFL learners’ burnout. Similarly, L2 grit has been suggested to effectively reduce FL learning burnout when other constructs such as teacher enjoyment [6], motivated behavior [39], and self-oriented learning perfectionism [40] were considered.

As a beneficial psychological trait, L2 grit has also been investigated in relation to FL achievement in previous studies. Some of these studies [41, 42] revealed a direct and significant effect of L2 grit on FL achievement, while others [43] failed to find significant relations between the two. Additionally, there were studies indicating no direct link between L2 grit and achievement, but only an indirect association mediated by emotions (e.g., enjoyment and anxiety), psychological constructs like personal best goals, and behavioral self-regulation [44–47]. Besides, building on the evidence that the two grit facets (i.e., COI and POE) play distinct roles in predicting academic outcomes [48], a few SLA studies explored the respective effects of L2 grit dimensions on FL achievement. For example, Zhao and Wang [49] demonstrated that POE and COI significantly influenced L2 achievement both directly and through the mediating effects of emotions. More studies [8, 44] reported that only POE was an influential predictor of FL achievement among EFL learners at secondary and university levels. Diverging from these findings, Li [50] and Sudina and Plonsky [51] showed that L2 achievement was significantly influenced by COI, with no significant impact observed for POE.

Drawing from the mixed evidence on the influence of L2 grit as well as the call to independently investigate the predictive effects of COI and POE within language-specific domains [51], the present study attempts to investigate the distinct contributions of COI and POE to FL achievement. Based on the demands-resources model, the study further examines whether FL learning burnout mediates the relationship between L2 grit components and FL achievement.

### FL achievement: academic outcome in the demands-resources model

Drawing on the demands-resources model in academic contexts, academic achievement has long been identified as an important academic outcome of student burnout. Extending the model’s energy-depleting process to FL learning, it is suggested that high demands and insufficient resources in FL learning increase EFL learners’ burnout risk, which may in turn lead to negative academic outcomes, in this case, lower FL achievement. Research in general education provides evidence for this negative academic burnout-achievement link [52] and supports the significant roles of all three burnout components (exhaustion, cynicism, and reduced efficacy; see Madigan and Curran [53] for a meta-analysis). Yet, evidence from FL learning remains relatively limited. Only a few studies investigated the relationship between FL learning burnout and achievement, and few explored the respective contributions of burnout dimensions. Among the existing studies, Ma et al. [2] investigated the mediating role of FL learning burnout in the link between teacher-student relationships and FL achievement, revealing that FL learning burnout negatively influenced FL achievement among Chinese secondary school students. Several studies with undergraduate EFL learners reported similar negative associations between the two constructs [17, 54, 55]. Building on prior evidence of a negative burnout-achievement link in EFL contexts, the present study attempts to advance this line of research by examining the respective effects of three FL learning burnout components on FL achievement among a relatively understudied group, namely, postgraduate EAP learners.

### The present study

The literature reviewed suggests that teacher emotional support and L2 grit can buffer FL learning burnout and enhance FL achievement. Moreover, the demands-resources model theoretically supports the relationship between these variables. According to the motivational process of the demands-resources theory, teacher emotional support and L2 grit can be regarded as environmental and personal resources helping EFL learners cope with demands in FL learning, which can effectively reduce their burnout, and ultimately improve their learning outcome. Hence, the present study hypothesizes that teacher emotional support and L2 grit positively predict FL achievement, and negatively predict FL learning burnout (Hypotheses 1–5). Concerning the association between FL learning burnout and achievement, prior literature indicates a negative relationship. The energy-depleting process of the model offers theoretical support to this relationship. According to the process, burnout resulting from high demands and stressors in FL learning undermines learner functioning and learning, leading to negative academic performance, such as lower FL achievement. Based on the empirical and theoretical evidence, we propose Hypothesis 6: FL learning burnout negatively predicts FL achievement. Taken together, the model also provides a clear rationale for FL learning burnout as a mediator between resources at contextual and personal levels (i.e., teacher emotional support and L2 grit) and learning outcome (i.e., FL achievement). Consequently, we propose that FL learning burnout negatively mediats the impact of teacher emotional support and L2 grit on FL achievement (Hypotheses 7–9).

**Fig. 1:**
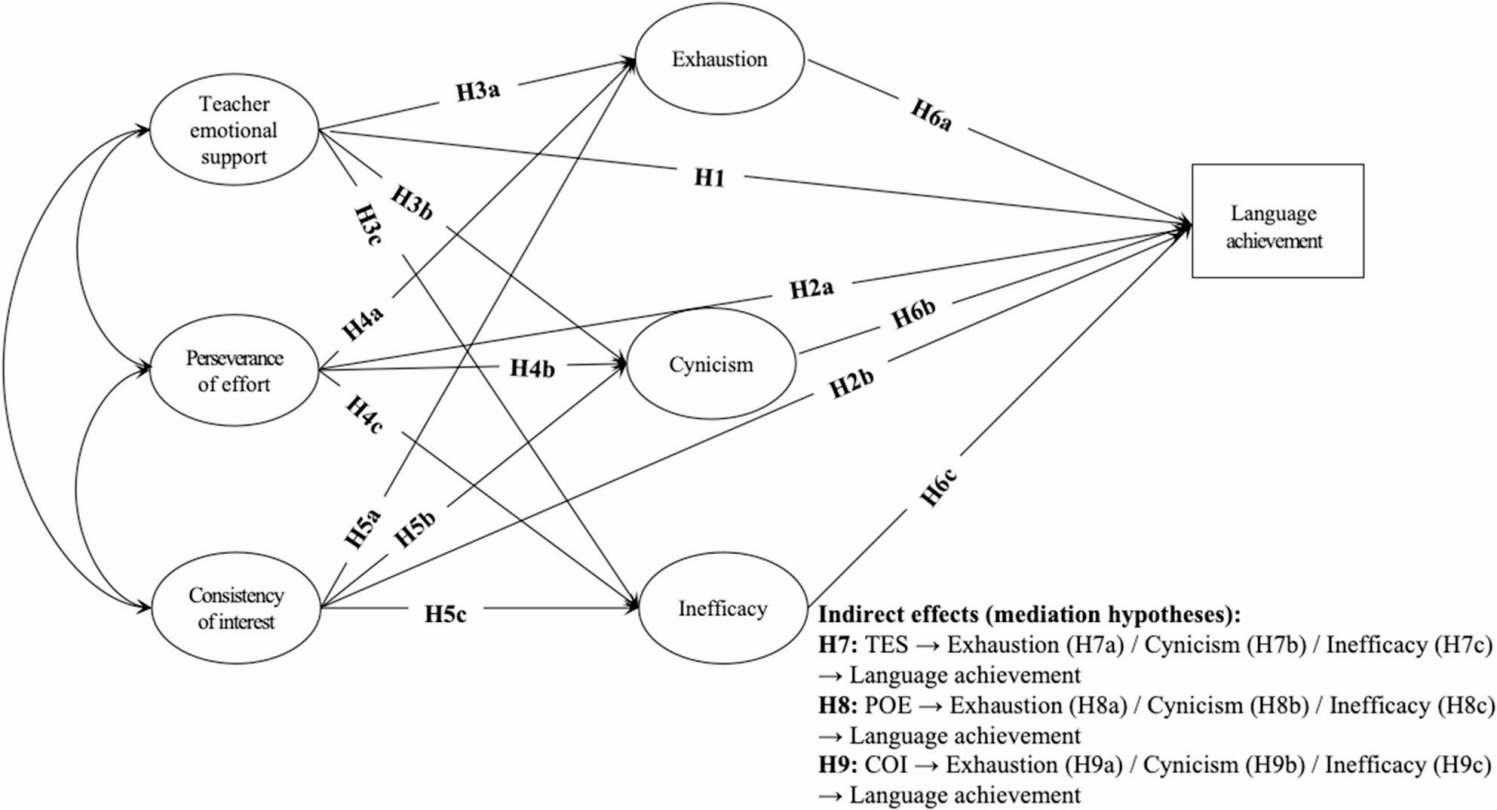
Hypothesized model of the present study

Moreover, empirical studies in general education indicated that academic burnout components exhibited differential relationships with study-related antecedents and outcomes [53, 56]. This suggests the need to investigate how FL learning burnout components (i.e., exhaustion, cynicism, and reduced efficacy) uniquely relate to FL learning resources and outcome. Similarly, the distinct roles of L2 grit dimensions (i.e., COI and POE) evidenced by previous studies in general education and FL learning [51, 57] underscore the importance to investigate how L2 grit dimensions are associated with other variables in the model. Accordingly, we proposed the following hypotheses, and a hypothesized model (see Fig. 1).

- H1: Teacher emotional support positively predicts FL achievement.
- H2 (H2a-H2b): Both L2 grit facets (i.e., POE and COI) positively predict FL achievement.
- H3 (H3a-H3c): Teacher emotional support negatively predicts components of FL learning burnout (i.e., emotional exhaustion, cynicism, and sense of inefficacy).
- H4 (H4a-H4c): POE, one facet of L2 grit, negatively predicts components of FL learning burnout.
- H5 (H5a-H5c): COI, the other facet of L2 grit, negatively predicts components of FL learning burnout.
- H6 (H6a-H6c): Components of FL learning burnout negatively predict FL achievement.
- H7 (H7a-H7c): Components of FL learning burnout mediate the effect of teacher emotional support on FL achievement.
- H8 (H8a-H8c): Components of FL learning burnout mediate the effect of POE on FL achievement.
- H9 (H9a-H9c): Components of FL learning burnout mediate the effect of COI on FL achievement.

## Methods

### Participants

Our participants were 1060 first-year graduate students from non-English disciplines at a comprehensive university in southeastern China, selected through convenience sampling. They were enrolled in an EAP (English for Academic Purposes) course titled *Academic Communication English*, a mandatory course in the first semester of their master’s program. Among them, 376 (35.5%) were females and 684 (64.5%) were males. They were between 19 and 31 years old, with a mean of 22.48 (SD = 1.00). They came from a wide range of academic backgrounds, representing more than 30 majors across disciplines such as humanities and social science, engineering and technology, and natural sciences. Most participants had been learning English for over 14 years. As for their English proficiency, 90.8% of them had passed CET-6 test, indicating an intermediate level of English proficiency.

### Instruments

The questionnaire was composed of a demographic survey (e.g., student ID, major, and gender) and three scales assessing perceived teacher emotional support, grit, and burnout in FL learning, all described in detail below. Student ID numbers were required so that the authors would be able to obtain learners’ final course grades. All scales were administered in Chinese to ensure clear understanding. Participants’ responses were recorded on a five-point rating scale, ranging from 1(*strongly disagree*) to 5 (*strongly agree*).

#### Perceived teacher emotional support

Participants’ perceived teacher emotional support was assessed using *EFL Learners’ Perceived Teacher Emotional Support Scale* proposed by Liu et al. [3]. The subscale is composed of 5 items, with items such as “*The English teacher treats us with respect.*” Prior studies [58] have shown that the scale is valid and suitable for Chinese university students. In the current sample, the Cronbach’s alpha for the teacher emotional support subscale was 0.935, demonstrating high internal consistency.

#### L2 grit

L2 grit was measured by Teimouri et al.’s [8] L2 Grit scale, which includes a 4-item COI subscale, with items such as “*I think I have lost my interest in learning English.*”, and a 5-item POE subscale with items like “*I am a diligent English language learner*”. COI items were reverse coded prior to data analysis, with higher scores representing stronger passion for L2 learning. The scale has been tested and found valid in several studies involving Chinese EFL learners [59]. In the present study, both subscales demonstrated acceptable reliability, with Cronbach’s alpha being 0.887 for POE and 0.793 for COI subscale.

#### Foreign language learning burnout

Maslach Burnout Inventory-Student Survey, originally developed by Schaufeli et al. [16] to measure students’ academic burnout, was adapted to assess English learning burnout. Expressions like “*a class*” and “*my studies*” were modified to more specific ones (i.e., “*an English class*” and “*my English studies*”) to better align with the research context. The scale contains 15 items measuring three dimensions of FL learning burnout: emotional exhaustion (5 items; e.g., *I feel emotionally drained by my studies.*), cynicism (4 items; e.g., *I doubt the significance of my English studies.*), and efficacy (6 items; e.g., *In my opinion*,* I am a good English learner.*). Items assessing efficacy were reverse coded before statistical analysis. The three sub-constructs in this study exhibited high reliability, with Cronbach’s α values of 0.941, 0.849 and 0.837, respectively.

#### Foreign language achievement

Learners’ course grades were adopted to assess their FL achievement. The final grades evaluate various aspects of academic English learning, including listening, speaking, reading, and writing in academic contexts. Course scores spanned from 0 to 100, with 100 as the highest score. A minimum score of 60 was required to pass the course.

### Data collection

Participants completed an online questionnaire distributed via *Wenjuanxing* at the end of the fall semester of 2023. Before administering the questionnaire, we explained the purpose of the survey, assured participants of the confidentiality of their responses, and clarified their right to choose whether to participate or not. They were also informed that providing their student ID numbers to access their final course grades was entirely voluntary. The questionnaire was then distributed to the students during scheduled class time, and it took them about 15 min to complete. Their final course grades were obtained from the department’s administrative staff at the beginning of the spring semester of 2024.

### Data analysis

Data were formatted and analyzed using SPSS 27.0 and Mplus 8. First, SPSS 27.0 was used to conduct normality tests, reliability, and Harman’s single-factor test, as well as descriptive and correlation analyses. Next, confirmatory factor analysis (CFA) was conducted using Mplus 8 to test the factor structure of each individual construct as well as the overall measurement model. Composite reliability (CR) and average variance extracted (AVE) were calculated to assess convergent validity, while maximum shared variance (MSV) and the Fornell-Larcker criterion [60] were used to evaluate discriminant validity. Subsequently, structural equation modelling (SEM) with maximum likelihood estimation was performed to examine the hypothesized model. The adequacy of both measurement and structural models was evaluated based on the following benchmark: CFI ≥ 0.90, TLI ≥ 0.90, RMSEA ≤ 0.08, and SRMR ≤ 0.08 [61]. Finally, mediating effects were examined using a bootstrap analysis based on 5,000 samples with bias-corrected 95% confidence intervals (CI). The mediating effect was considered significant when the bias-corrected 95% CI did not include zero.

## Results

### Preliminary analyses

To ensure that the data were suitable for SEM analysis, several preliminary tests were conducted. First, since the independent and mediating variables were measured using the same self-report questionnaire, Harman’s single-factor test was performed to examine the potential presence of common method bias. Results showed that six factors with eigenvalues greater than 1 were extracted, and the first factor accounted for 34.8% of the total variance, which was below the commonly used threshold of 50% [62, 63]. These results suggest that common method bias was unlikely to be a serious concern in the present study. Second, data normality was assessed. As shown in Table 1, the absolute skewness and kurtosis values of each variable were below the thresholds of 3 and 10 respectively, suggesting no substantial departure from normality [64].

**Table 1:** Descriptive statistics of variables

	TES	POE	COI	Exhaustion	Cynicism	Inefficacy	FLA
Mean	4.15	3.07	3.17	2.85	2.49	2.69	84.2
SD	0.64	0.69	0.74	0.94	0.85	0.60	4.26
Skewness	-0.89	-0.06	0.20	0.21	0.46	0.37	-0.63
Kurtosis	2.33	0.64	0.18	-0.25	0.27	0.63	1.06

*N *= 1,060

*TES* teacher emotional support, *POE* perseverance of effort, *COI* consistency of interest, *FLA* foreign language achievement

Results for descriptive and correlation analyses were presented in Tables 1 and 2, respectively. Participants reported a comparatively high level of perceived teacher emotional support (M = 4.15, SD = 0.64), a moderate level of both facets of grit (M_POE_ = 3.07, SD_POE_ = 0.69; M_COI_ = 3.17, SD_COI_ = 0.74), and a relatively low level of emotional exhaustion, cynicism as well as feeling of inefficacy (M_Exhau_ = 2.85, SD_Exhau_ = 0.94; M_Cyn_ = 2.49, SD_Cyn_ = 0.85; M_Ineff_ = 2.69, SD_Ineff_ = 0.60). The mean score of their course grade was 84.20 (SD = 4.26). Pearson correlation analysis revealed significant correlations among all variables (see Table 2). Teacher emotional support, POE, COI, and FL achievement showed positive correlations with one another (*r* ranging from 0.066 to 0.651), while the three components of burnout were negatively associated with other variables (*r* ranging from − 0.093 to − 0.585).

**Table 2:** Correlation analysis among variables

	1	2	3	4	5	6
1 TES	-	-	-	-	-	-
2 POE	0.238**	-	-	-	-	-
3 COI	0.173**	0.363**	-	-	-	-
4 Exhaustion	− 0.302**	− 0.339**	− 0.485**	-	-	-
5 Cynicism	− 0.261**	− 0.392**	− 0.585**	0.651**	-	-
6. Inefficacy	− 0.277**	− 0.516**	− 0.335**	0.494**	0.449**	-
7 FLA	0.066*	0.133**	0.070*	− 0.183**	− 0.093**	− 0.263**

*N* = 1,060

*TES* teacher emotional support, *POE* perseverance of effort, *COI* consistency of interest, *FLA* foreign language achievement

**p < 0.01, *p < 0.05

### Reliability, validity, and measurement model evaluation

Prior to testing the overall measurement model, CFA was conducted for each individual scale to examine its factor structure, and Cronbach’s alpha was calculated to assess internal consistency reliability. As shown in Table 3, all individual scales demonstrated acceptable internal consistency and adequate model fit. Next, the overall measurement model comprising all six latent variables was tested. Results revealed that the overall measurement model fit the data well: χ^2^/df = 4.55; CFI = 0.941; TLI = 0.933; RMSEA = 0.058 [0.055, 0.061]; SRMR = 0.055. Furthermore, convergent validity was assessed through CR and AVE. As reported in Table 4, CR values ranged from 0.822 to 0.942, all above the recommended benchmark of 0.7, and AVE values ranged from 0.513 to 0.765, all exceeding the 0.5 threshold [65], thereby supporting convergent validity. Discriminant validity was evaluated using the Fornell-Larcker criterion and MSV values. According to Fornell and Larcker [60], discriminant validity is supported when the square root of AVE for a certain construct exceeds its correlations with all other constructs. Results in Table 4 showed that the square root of each construct’s AVE (bold diagonal values) exceeded all inter-construct correlations (off-diagonal values), and MSV of each construct was lower than its corresponding AVE, together providing evidence for satisfactory discriminant validity.

**Table 3:** Cronbach’s α and CFA Model fit indices for all variables (*N* = 1060)

Construct	Cronbach’s α	χ²/df	CFI	TLI	RMSEA [90%CI]	SRMR
TES	0.935	2.21	0.999	0.996	0.041[0.013, 0.070]	0.006
POE	0.887	3.46	0.995	0.983	0.047[0.029, 0.079]	0.011
COI	0.793	2.68	0.996	0.976	0.054[0.027, 0.082]	0.011
Exhau	0.941	4.03	0.997	0.988	0.053[0.045, 0.075]	0.007
Cyn	0.849	1.72	0.998	0.978	0.026[0.010, 0.081]	0.003
Ineff	0.837	3.34	0.990	0.974	0.052[0.041, 0.074]	0.021

*TES* teacher emotional support, *POE* perseverance of effort, *COI* consistency of interest, *Exhau* exhaustion, *Cyn* cynicism, *Ineff* inefficacy

**Table 4:** Convergent and discriminant validity of measurement model

	CR	AVE	MSV	Fornell-Larcker					
				TES	POE	COI	Exhaustion	Cynicism	Inefficacy
TES	0.940	0.761	0.091	**0.872**	-	-	-	-	-
POE	0.888	0.612	0.361	0.236	**0.783**	-	-	-	-
COI	0.822	0.539	0.520	0.234	0.461	**0.734**	-	-	-
Exhau	0.942	0.765	0.527	-0.301	-0.367	-0.586	**0.875**	-	-
Cyn	0.870	0.628	0.527	-0.252	-0.476	-0.721	0.726	**0.792**	-
Ineff	0.862	0.513	0.361	-0.293	-0.601	-0.453	0.554	0.531	**0.716**

*TES* teacher emotional support, *POE* perseverance of effort, *COI* consistency of interest, *Exhau* exhaustion, *Cyn* cynicism, *Ineff* inefficacy

The square root of AVE is demonstrated along the diagonal line in bold

### Test of hypothesized model

**Fig. 2:**
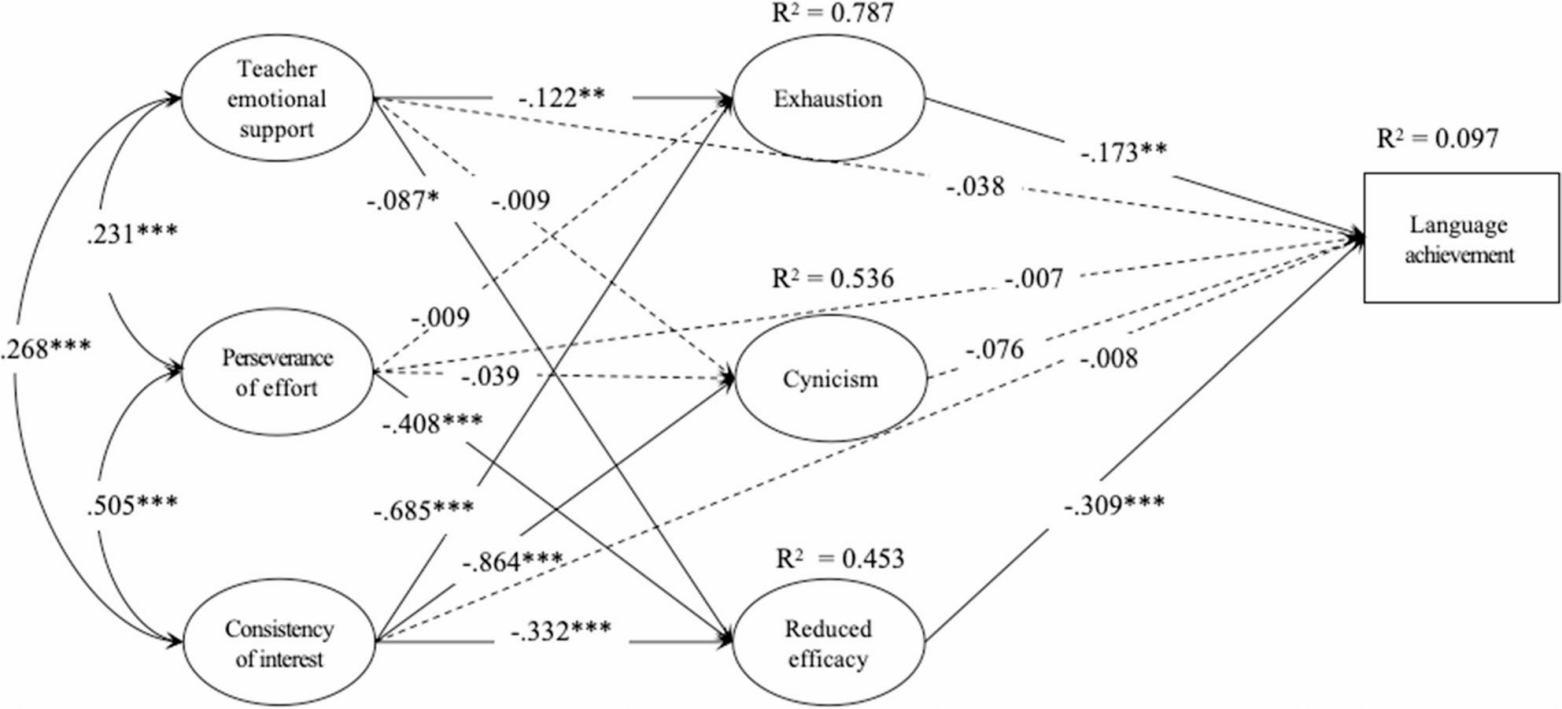
Structural model with standardized coefficients. Note: *p < 0.05; **p < 0.01; ***p < 0.001

The hypothesized relationships among the variables were examined using SEM. The proposed model demonstrated a satisfactory fit to the data: χ^2^/df = 4.96; CFI = 0.930; TLI = 0.920; RMSEA = 0.061[0.058, 0.064]; SRMR = 0.059. Figure 2 displayed all pathways in the model and their standardized coefficients, with solid lines indicating statistically significant relationships and dashed lines indicating non-significant ones. SEM results showed that teacher emotional support, COI, and POE did not directly influence FL achievement (β_TES_ = − 0.038, *p* > 0.05; β_POE_ = − 0.007, *p* > 0.05; β_COI_ = − 0.008, *p* > 0.05). Teacher emotional support significantly predicted exhaustion (β = − 0.122, *p* < 0.01) and inefficacy (β = − 0.087, *p* < 0.05), but not cynicism (β = − 0.009, *p* > 0.05). POE negatively predicted inefficacy (β = − 0.408, *p* < 0.001), but not the other two indicators of burnout (β = − 0.009, *p* > 0.05; β = − 0.039, *p* > 0.05, respectively for exhaustion and cynicism), while COI served as a negative predictor of all three burnout components (β = − 0.685, *p* < 0.001; β = − 0.864, *p* < 0.001; β = − 0.332, *p* < 0.001; respectively for exhaustion, cynicism, and inefficacy). In total, the model explained 78.7% of the variance in exhaustion, 53.6% in cynicism, and 45.3% in reduced efficacy. As for the effect of burnout on FL achievement, sense of inefficacy emerged as the strongest predictor (β = − 0.309, *p* < 0.01), followed by emotional exhaustion (β = − 0.173, *p* < 0.01), whereas cynicism failed to significantly predict FL achievement (β = − 0.076, *p* > 0.05).

Bias-corrected bootstrap tests with 5000 samples were conducted to examine the mediating role of FL learning burnout. The results of indirect effects were shown in Table 5. A 95% CI excluding 0 indicated a statistically significant indirect path. As seen in the table, teacher emotional support indirectly contributed to FL achievement via the mediating role of both inefficacy (β = 0.027, 95% CI: [0.005, 0.055]) and exhaustion (β = 0.021, 95% CI: [0.006, 0.045]). As for the indirect effect of grit, POE significantly improved FL achievement by reducing learners’ sense of inefficacy (β = 0.126, 95% CI: [0.083, 0.177]), while COI indirectly influenced English achievement through the mediating effect of exhaustion as well as inefficacy (β_Exhau_ = 0.119, 95% CI: [0.050, 0.198]; β_Ineff_ = 0.103, 95% CI: [0.063, 0.156]). However, all paths involving cynicism as a mediator were not significant, nor was the path from POE to exhaustion to FL achievement. When comparing their effects on FL achievement, the magnitude was largest for COI (β = 0.222), followed by POE (β = 0.126), and smallest for teacher emotional support (β = 0.048). R^2^ values indicated that the model accounted for 9.7% of the variance in FL achievement.

**Table 5:** Direct and indirect effects in the structural model

Pathways	β	SE	Z	*P*	BCa 95% CI	
					Lower	Upper
Direct effects						
TES→LA	-0.038	0.032	-1.172	0.241	-0.100	0.026
POE→LA	-0.007	0.044	-0.162	0.872	-0.094	0.078
COI→LA	-0.008	0.129	-0.061	0.952	-0.255	0.258
Indirect effects						
**TES→Exhau→FLA**	**0.021**	**0.009**	**2.234**	**0.025**	**0.006**	**0.045**
TES→Cyn→FLA	0.001	0.008	0.086	0.931	-0.012	0.021
**TES→Ineff→FLA**	**0.027**	**0.012**	**2.154**	**0.031**	**0.005**	**0.055**
POE→Exhau→FLA	0.002	0.009	0.179	0.858	-0.016	0.021
POE→Cyn→FLA	0.003	0.010	0.296	0.767	-0.013	0.028
**POE→Ineff→FLA**	**0.126**	**0.024**	**5.211**	***p*** **<** **0.001**	**0.083**	**0.177**
**COI→Exhau→FLA**	**0.119**	**0.037**	**3.229**	**0.001**	**0.050**	**0.198**
COI→Cyn→FLA	0.065	0.101	0.650	0.516	-0.141	0.257
**COI→Ineff→FLA**	**0.103**	**0.024**	**4.341**	***p*** **<** **0.001**	**0.063**	**0.156**

β refers to standardized coefficients

Significant pathways are in bold

*TES* teacher emotional support, *Exhau* emotional exhaustion, *Cyn* Cynicism, *Ineff* reduced efficacy, *POE* perseverance of effort, *COI* consistency of interest, *FLA* foreign language achievement

## Discussion

### Teacher emotional support and L2 grit as predictors of FL achievement

Contrary to our expectations, neither teacher emotional support nor L2 grit directly predicted FL achievement, rejecting Hypotheses 1 and 2. Instead, both influenced achievement only indirectly through burnout, partially supporting Hypotheses 7, 8, and 9. This indirect pattern is consistent with the motivational process in the demands-resources model, which posits that resources enhance positive outcomes by buffering the harmful effects of demands on burnout [34]. This suggests that, for this population, teacher emotional support and L2 grit, as environmental and personal resources, may have contributed to FL achievement primarily by alleviating the psychological strain that impedes learning, rather than by directly enhancing the linguistic processes underlying FL performance.

The indirect link between teacher emotional support and FL achievement via FL learning burnout aligns with previous studies in general education [66, 67] and foreign language education [4, 36], which reported that emotional support from teachers failed to influence students’ academic or language achievement directly, but worked in an indirect manner via students’ internal factors, such as self-esteem, buoyancy, emotions, and engagement. The consistent absence of a direct effect across these studies, despite differences in context and mediating constructs, suggests that teacher emotional support may function primarily as a contextual resource that shapes the psychological conditions for learning, by reducing burnout and preserving emotional energy, rather than directly influencing FL achievement. This may not be surprising given that teacher emotional support is a resource targeted at learners’ affective states rather than their linguistic processing. Its influence on achievement may therefore need to be channeled through psychological mechanisms, such as reduced burnout, before it is reflected in learning outcomes.

When compared to L2 grit components, it showed the smallest effect on students’ FL achievement. The relatively weak influence of teacher emotional support may reflect that the support students received from teachers did not align well with their needs, exerting limited effect on buffering their negative emotional experiences, attitudes, and beliefs. This interpretation is supported by the findings of Wang et al. [68], who found that among Chinese university students in AI-assisted EFL learning contexts, only one of the three identified learner groups (i.e., comfort-seeking learners) strongly prioritized teacher emotional support, while the other two profiles placed less emphasis on affective reassurance and valued pedagogical structure or hands-on technical guidance. This suggests that the need for teacher emotional support may be particularly salient for only a subgroup of learners rather than being universally salient, which may partly account for its limited overall effect on FL achievement in the present study. Additionally, the comparatively weaker influence of teacher emotional support may partly be attributed to the characteristics of our postgraduate sample. Having developed greater academic maturity through years of formal education, these learners may be more inclined to draw on internal resources, such as grit, than on external emotional support from teachers. This is consistent with evidence that as learners advance on their education, internal motivational and self-regulatory resources become increasingly dominant, while environmental factors, including teacher-related support, play a diminishing role [69, 70], which may weaken the relative contribution of teacher emotional support to FL achievement in this population.

The same indirect-only pattern was revealed for L2 grit, supporting the assumption in the demands-resources model that personal resources influence learning outcome primarily through enhancing engagement or buffering burnout, rather than directly. The finding resonates with studies in both FL education [44–46] and broader academic contexts [71], which reported that grit did not directly predict achievement but rather indirectly through internal psychological processes such as academic emotions, beliefs, and engagement. These findings indicate that grit serves less as a direct driver of FL achievement and more as a psychological buffer that helps learners cope with the demands of FL learning. Rather than improving FL achievement directly, grit may contribute to it by protecting learners from the emotional strain of demanding FL tasks, preserving the psychological resources that would otherwise be depleted by burnout.

Regarding the independent contributions of the two grit components, COI demonstrated a larger effect on FL achievement through its impact on emotional exhaustion and reduced efficacy, followed by POE via reduced efficacy. The finding is inconsistent with studies identifying POE as a stronger predictor of academic or language achievement [48, 49], but aligns with other studies reporting a stronger role of COI [50, 51]. This inconsistency across studies may reflect both methodological and contextual differences. One possible explanation lies in the different measures used to assess L2 grit. Our finding is consistent with studies using the same L2 grit instrument [8] as the present study, but differs from those using the general grit scale [72]. This further underscores the importance of employing a language-specific grit measure in our field. Additionally, we speculate that certain demographic factors related to being a postgraduate student in the Chinese educational system may help explain the difference in findings. Secondary school and undergraduate students in China strive to pass university entrance exams or national English tests like CET-4 and CET-6. By contrast, postgraduate students are generally free from these external pressures. Thus, their engagement with English learning is more likely to be driven by intrinsic interest. Given that they no longer need to invest as much effort into English learning, continuous interest may instead be a more important factor at this educational stage. In other words, for advanced learners who face fewer external pressures to learn English, continuous interest may become a more influential resource in sustaining engagement and buffering against burnout, potentially because intrinsic curiosity compensates for the reduced role of external motivational drivers.

### Teacher emotional support and grit as predictors of burnout

Teacher emotional support, as an external resource, was inversely associated with students’ emotional exhaustion and inefficacy (supporting H3a and H3c), but not significantly with cynicism (rejecting H3b). The protective effect against emotional exhaustion is not surprising. Emotional exhaustion manifests as a state of being overwhelmed and depleted of emotional resources [73]. Teacher emotional support, by attending to learners’ emotional needs, may help replenish emotional resources and ease the feeling of being overwhelmed by high study demands, thereby fostering greater resilience against exhaustion [5, 74]. Similarly, the buffering effect on inefficacy is consistent with prior research linking teacher emotional support to enhanced self-efficacy [75, 76]. Through encouragement, empathy, and positive feedback, emotionally supportive teachers may strengthen individuals’ confidence in their ability to manage challenges, which in turn empowers them to invest greater effort and persist through difficulties, ultimately strengthening their academic skills and sense of efficacy [77–79].

However, in contrast to our initial hypothesis, teacher emotional support failed to predict cynicism in the current sample. Cynicism in the burnout literature is understood as a defensive withdrawal from one’s studies, characterized by detachment and a loss of perceived meaning [73]. Unlike emotional exhaustion and inefficacy, which reflect depletion of emotional resources and confidence that emotional support can plausibly replenish, cynicism may be more closely tied to cognitive and motivational processes, specifically, learners’ appraisals of the value and relevance of language learning to their personal and professional goals. This is congruent with Söderholm et al.'s [56] argument that while an emotionally supportive environment may help buffer against students’ feelings of exhaustion and inefficacy, cynicism appears to be more strongly driven by internal cognitive factors such as goal orientation and attitude towards learning. Because these deeper attitudinal appraisals concern the perceived meaningfulness and purpose of one’s studies rather than the availability of emotional resources, they may not be influenced by teacher emotional support alone, which may help explain the non-significant relationship in the present study.

Turning to L2 grit, COI significantly predicted all dimensions of burnout (supporting H5a, H5b, and H5c), while POE was only a significant predictor of reduced efficacy (supporting H4c, rejecting H4a and H4b). The result concurs with the broader literature on the protective effect of grit on academic burnout [6, 80], but extends it by suggesting that the two grit facets may function through distinct mechanisms. The demands-resources model posits that personal resources help individuals cope with demands and reduce burnout [34], but it does not specify whether all resources should buffer all burnout dimensions equally. Our findings suggest that the scope of a resource’s protective effect may depend on the particular psychological function it serves, which is consistent with recent calls within the demands-resources literature for greater attention to the specific roles and functions of different resources [34, 81]. The negative associations between COI and all three burnout dimensions may be understood in terms of the kind of engagement it sustains. COI, as a resource reflecting enduring passion for and curiosity about language learning, may help learners maintain a sense of personal meaning and psychological investment in their studies [8, 82], which may protect against burnout at multiple levels. Emotionally, it may help learners experience demanding tasks as worthwhile rather than merely burdensome, which could help preserve emotional energy and reduce susceptibility to exhaustion [34]. In terms of cynicism, it may maintain a sense of personal connection to and perceived value in language learning, countering the detachment characteristic of cynicism. In terms of efficacy, it may sustain deeper cognitive engagement with learning tasks, which facilitates more effective skill development and in turn helps learners maintain a sense of efficacy [83], thereby buffering against feelings of inadequacy.

POE, by contrast, predicted only reduced efficacy, and this narrower effect is consistent with the nature of the resource. Sustained effort in language learning contributes to the accumulation of skills and knowledge, which strengthens learners’ sense of control over academic tasks and reinforces their confidence in their own competence [77]. This pathway from effort to mastery experiences offers a plausible account of why POE specifically mitigates feelings of inadequacy. However, the non-significant associations between POE and the other two burnout dimensions suggest that behavioral persistence, while valuable, may not be sufficient on its own to address the emotional and attitudinal aspects of burnout. Persistent effort does not necessarily replenish the emotional resources that are depleted under sustained demands, nor does it, in the absence of complementary motivational or affective supports, inherently restore a sense of personal meaning or relevance in language learning.

The finding that COI outweighs POE in predicting burnout at this stage of language learning is particularly noteworthy, as it diverges from prior research suggesting POE as a stronger predictor of academic and language learning [48, 49]. Although somewhat unexpected, the result was consistent with Sudina and Plonsky's [51] finding that COI exhibited stronger association with language-related academic learning than POE, and with their argument that demographic and cultural factors may explain the contradicting findings. Building on this, we suggest that the relative salience of each grit facet in buffering burnout may partly depend on the broader motivational context in which learning takes place. In our study, it is possible that postgraduate learners who have already developed considerable linguistic competence and face less external pressure to learn English rely less on sustained effort and more on sustained interest to remain engaged with language learning, thereby making COI a more salient predictor of burnout at this educational stage. Though this finding might not hold true for all EFL populations, it highlights the importance of cross-validating the impact of POE and COI across diverse samples, suggesting that the idea to focus solely on POE in subsequent studies on grit [57] may be premature.

### Burnout as a predictor of FL achievement

Among the three burnout components, only emotional exhaustion and reduced efficacy significantly predicted EFL learners’ achievement, whereas cynicism did not, supporting H6a and H6c but not H6b. The result partially diverged from Madigan and Curran’s [53] meta-analytic finding that all three burnout dimensions significantly and negatively predict academic achievement in general education. This partial divergence suggests that the relative predictive salience of individual burnout components may vary depending on academic domains, the sociocultural contexts, and the characteristics of the learner population under investigation.

The most salient finding regarding the burnout-achievement relationship in this study might be that reduced efficacy, among the three burnout components, had the largest effect on EFL learners’ language achievement. This aligns with a robust body of literature underscoring the crucial role of self-efficacy in academic success [84], as well as previous burnout studies identifying inefficacy as the strongest negative predictor of academic achievement [16, 53]. From a social-cognitive perspective, Bandura [85] noted that individuals with low self-efficacy tend to attribute inadequate performance to limited ability rather than insufficient effort, and that such disbelief in their own abilities may lead them to avoid challenging activities and withdraw effort when difficulties arise, eventually producing behavioral patterns that reinforce these negative self-beliefs. This is consistent with research showing that students with low self-efficacy are more likely to reduce their use of effective learning strategies in the face of setbacks and to avoid challenging tasks [86, 87], both of which can limit meaningful engagement and ultimately lower academic achievement.

Another effective predictor in the present sample was emotional exhaustion, which concurs with a substantive body of research reporting its adverse effect on learning performance [88, 89]. This finding can be understood through the lens of resource depletion. When emotional resources are depleted or threatened with depletion, individuals tend to reduce their effort and commitment devoted to stressful events as a strategy for conserving resources [90], which subsequently leads to lower academic achievement. Beyond this resource conservation mechanism, emotional exhaustion has also been associated with impaired cognitive functioning, maladaptive coping strategies, as well as reduced self-regulatory behaviors in learning process [88, 91], which may further decrease their odds of success in their academic endeavors. In the context of EFL learning, where sustained engagement across receptive and productive skill domains places continuous demands on learners’ cognitive and affective resources, these effects may be particularly detrimental to FL achievement.

However, contradicting our hypothesis, cynicism did not predict language learners’ achievement at a significant level. This is inconsistent with studies in other academic domains revealing a significant cynicism-achievement link [52, 92]. Based on a meta-analysis of 22 studies, Madigan and Curran [53] reported a small-to-medium but robust average effect size of cynicism (*r* = − 0.24), suggesting that a skeptical attitude towards studying causes students to withdraw and disengage from their studies, ultimately lowering their achievement. The unexpected finding of our study might be attributed to several reasons. At the contextual level, the negative impact of cynicism may be weakened by Chinese cultural values, including collectivism, respect for authority, and long-term orientation [93]. In a collectivist culture, individuals tend to prioritize group objectives over individual ones. Our participants, engaged in semester-long teamwork, likely invested time and effort in language learning to meet collective objectives, despite any personal detachment. Additionally, influenced by the value of respect for authority, Chinese students are more likely to comply with teachers, school structure, and regulations, hence demonstrating greater tolerance for required tasks, even those without personal endorsement. Long-term orientation, another value embedded in Chinese culture, promotes perseverance and effort in education, making Chinese students more willing to work hard in the present to prepare for the future [94]. Taken together, these cultural orientations may have mitigated the harmful effect of a cynical attitude by sustaining behavioral effort and engagement even when attitudinal commitment to language learning has diminished. At the individual level, the relatively advanced English proficiency of the participants may also be relevant. It is plausible that advanced and skilled language learners have developed established study routines and learning strategies that are sufficient to sustain performance even in the presence of cynical attitudes toward their studies. In other words, for these learners, the negative link between cynical attitudes and achievement may be partially weakened by well-established learning habits. In sum, these findings underscore the importance of addressing their burnout experience, especially their emotional exhaustion and sense of reduced efficacy, as these two dimensions appear to play a more influential role in shaping language learning outcomes in this population.

### Burnout as a mediator

The mediation analyses revealed that emotional exhaustion and reduced efficacy, but not cynicism, served as significant mediators in the relationships between teacher emotional support, L2 grit, and FL achievement. These differentiated mediation patterns suggest that the three burnout dimensions may not contribute equally to the demands-resources pathways in FL learning, but instead play distinct roles in linking resources to learning outcomes.

The mediating role of emotional exhaustion may be interpreted through the health impairment pathway of the demands-resources framework, which posits that insufficient resources make learners vulnerable to chronic energy depletion under sustained academic demands [81]. In the present study, emotional exhaustion mediated the influence of teacher emotional support and the COI facet of grit, but not POE, on FL achievement. This pattern may suggest that emotional exhaustion is more sensitive to resources that are affective in nature. Teacher emotional support may shape learners’ emotional experience by providing empathy, care, and a sense of psychological safety, whereas sustained passion (i.e., COI) can help learners maintain an enduring emotional investment in language learning over time. In this sense, both resources may protect learners against emotional depletion by supporting the emotional dimension of their learning experience. The absence of emotional exhaustion as a mediator for POE is also plausible. POE is more closely associated with behavioral persistence in the face of difficulty, and thus may reflect learners’ ability to continue investing effort despite fatigue, rather than a resource that targets at emotional needs and alleviates emotional depletion. In other words, perseverant learners may be able to keep performing despite emotional strain, rather than reducing the strain itself, which would explain why perseverance does not operate through the emotional exhaustion pathway.

Reduced efficacy emerged as the most prominent mediator as it transmits the effects of all three predictors (i.e., teacher emotional support, POE, and COI) on FL achievement. That all three resources converged on reduced efficacy as a common mediating mechanism, regardless of whether the resource was contextual or personal, affective or behavioral, suggests that learners’ confidence in their own competence functions as a central channel through which diverse resources shape FL achievement. Within the demands-resources framework, these mediation paths reflect the direct negative influence of resources on burnout, an empirically established link recognized in the model [81], whereby resources not only foster engagement through the motivational pathway but also directly reduce burnout components that would otherwise impair performance. Compared with emotional exhaustion, which mediated only two of the three pathways, reduced efficacy appears to play a broader role, suggesting that the erosion of competence beliefs may be a more influential mechanism than energy depletion in transmitting the influence of resources onto FL achievement. This extends current understanding of how resources relate to academic burnout in FL learning. They protect FL achievement not solely by preserving learners’ emotional energy, but also, and perhaps more broadly, by sustaining learners’ appraisal of their own competence. This finding also resonates with the broader positive psychology perspective, which emphasizes that optimal functioning depends substantially on the presence of positive psychological resources such as perceived competence, rather than merely on the absence of negative affective states [14]. From this perspective, protecting and strengthening learners’ sense of competence may be at least as important as alleviating emotional exhaustion in promoting FL achievement.

Cynicism did not function as a significant mediator in any resource-achievement pathway, though the reasons differed across resources. Teacher emotional support and POE did not significantly influence cynicism, likely because cynicism reflects an attitudinal judgement about the perceived value and relevance of language learning rather than an affective or behavioral state. Teacher emotional support targets learners’ emotional needs, whereas POE sustains behavioral effort in the face of difficulty. However, neither appears to address learners’ deeper appraisals of meaning and purpose that underlie cynical withdrawal. COI, by contrast, did significantly predict cynicism, suggesting that sustained intrinsic interest in language learning may help protect against attitudinal withdrawal because it keeps learners engaged at the level of personal meaning and perceived value, a dimension closely related to cynicism. Nevertheless, even when reduced by COI, lower levels of cynicism did not translate into higher FL achievement in this sample. As discussed earlier, cultural values emphasizing collectivism and respect for authority, together with well-established study routines among these advanced learners, may have sustained adequate performance regardless of learners’ detached attitude towards language learning, thereby weakening the link between cynicism and achievement. These patterns suggest that cynicism may function differently from emotional exhaustion and reduced efficacy in the resources-outcome pathway. On the one hand, it appears to be responsive to a narrower range of resources, particularly those that engage learners’ sense of purpose rather than their emotional states or behavioral habits. On the other hand, even when reduced, its association with achievement may vary across contexts and populations.

Taken together, these differentiated mediation patterns underscore that burnout does not function as a single process in the resource-achievement relationship. Each dimension operates through a distinct pathway, with reduced efficacy serving as the most consistent mediator, emotional exhaustion responding selectively to affective resources, and cynicism responsive only to resources that engage learners’ sense of purpose yet largely disconnected from achievement outcomes in this context. These findings point to the importance of adopting a dimension-specific perspective on burnout in both future research and pedagogical practice in FL learning.

### Limitations and future research

The current study is constrained by several limitations. First, given its cross-sectional design, the study cannot establish causal relationships between the variables. Longitudinal studies can be conducted to further clarify causal and long-term relationships. Second, focusing on Chinese postgraduate EAP learners from one university limits the generalizability of our findings. Diverse sampling methods should be adopted to recruit a broader range of participants across different contexts in future studies. Third, the present study relied solely on participants’ self-reports, which may have been influenced by social desirability. Further investigations can consider a mixed-methods approach, incorporating qualitative data to allow for data triangulation. Finally, the present study adopted students’ course grade as an indicator of language achievement. Future research may consider employing standardized language proficiency tests to obtain a more nuanced assessment.

The results of this study can nevertheless offer some new insights for SLA researchers interested in advancing positive psychology in language learning. First and foremost, the large effect size of L2 grit suggests that its role in impacting L2 burnout and achievement needs a lot more study. Second, COI was proven to exert the greatest effect on FL learning burnout and achievement. The lack of studies tackling interest as a variable is noticeable in SLA. Future research should therefore investigate interest as an independent positive construct in L2 learning, for example, its developmental trajectories over time and how to support it at different developmental stages [82]. Lastly, the direct pathway between burnout and language achievement uncovered in the present study suggests the need to deploy a rigorous research program on L2 burnout, including in-depth investigations of its effects on various aspects of L2 learning as well as intervention studies that lead the way to offering effective coping strategies to language learners.

### Pedagogical implications

The findings of the present study can nevertheless offer some practical implications for language teachers and learners. First, as both grit components emerged as strong predictors of FL learning burnout and achievement, with COI showing greater influence, teachers should give deliberate pedagogical attention to sustaining learners’ intrinsic interest. Rather than relying solely on external incentives such as grades or course requirements, teachers can nurture learners’ interest by connecting learning materials to their disciplinary interests and professional goals, incorporating personally meaningful topics and tasks, and offering autonomy in task selection where possible. For EAP learners specifically, aligning language instruction with learners’ academic disciplines, for example, through content-based or project-based approaches, may help learners sustain the sense of personal relevance, which could in turn support sustained interest over time. To strengthen students’ perseverance, teachers can support them by facilitating their goal setting, action planning, and progress monitoring [95].

Second, the significant role of teacher emotional support across multiple pathways suggests that how teachers relate to learners emotionally matters beyond the instructional content they deliver. However, the relatively modest overall effect, considered alongside evidence that teacher emotional support may be particularly salient for only certain learner profiles [68], suggests that a differentiated approach may be more effective than a uniform one. Instead of providing emotional support in a uniform way, teachers might identify students who are most in need of emotional scaffolding and allocate such support accordingly, while providing other learners with the structured pedagogical or technical guidance they may prioritize.

Third, the significant mediating role of burnout, specifically reduced efficacy and emotional exhaustion, points to the need to address burnout-related barriers to FL achievement. Given that reduced efficacy emerged as the most prominent mediator linking resources to achievement, teachers should place a central emphasis on fostering learners’ sense of competence. Teachers can achieve this by setting incremental and attainable learning goals, providing feedback that highlights progress rather than deficits, and designing tasks that are challenging yet within learners’ reach, so that learners accumulate experiences of mastery that counteract the erosion of efficacy beliefs. For advanced EAP learners such as postgraduate students in this study, this could mean breaking complex academic tasks such as literature discussions or discipline-specific writing into manageable steps that allow mastery at each stage. Meanwhile, the significant mediating role of emotional exhaustion highlights the need to attend to learners’ emotional states alongside their sense of competence. While sustaining learners’ interest and providing emotional support, as discussed above, may help alleviate emotional exhaustion, teachers should also be attentive to signs of emotional depletion, such as growing disengagement, fatigue, or withdrawal. Practical steps might include pacing demanding tasks to avoid sustained pressure, building in opportunities for recovery, and creating a classroom climate in which learners feel comfortable expressing difficulties.

## Conclusion

Grounded in positive psychology and the demands-resources model [9, 10], the present study investigated the intricate interplay among contextual and individual resources (i.e., teacher emotional support and L2 grit, respectively), FL learning burnout, and FL achievement among Chinese postgraduate EAP learners. First, teacher emotional support and L2 grit contributed to EFL learners’ language achievement, but only indirectly through burnout components, with magnitudes that were weak for the teacher support variable but large for L2 grit. Second, COI (the stronger facet of grit in the current study) was essential in mitigating burnout in language learning: it greatly mitigated cynicism (one of the three burnout components), but also exhaustion and, to a lesser extent, reduced efficacy, while POE did not alleviate exhaustion or cynicism, but substantially impacted reduced efficacy. Conversely, teacher emotional support, though significantly related to exhaustion and reduced efficacy, contributed minimally to mitigating both aspects. Third, regarding the predictive effect of burnout on FL achievement, it was only reduced efficacy and exhaustion, but not cynicism, that directly affected FL achievement. The study contributes to the current SLA literature in positive psychology by offering a holistic view on how the combination of positive traits (i.e., grit), emotional components (i.e., burnout), and learning environments (i.e., teacher emotional support) interact and link to FL achievement. In particular, the differentiated mediating mechanisms revealed in this study, where different resource types operated through distinct burnout components with varying magnitudes, extend our understanding of the demands-resources model in the EFL learning context. The study also expands our understanding of grit and burnout in FL learning by demonstrating the independent and distinct effects of their respective components. Lastly, the study diversifies the participant sample and reduces sampling bias in positive psychology research in SLA by focusing on postgraduate EAP learners, an important yet underexplored learner population.

## Data Availability

Data used in this study are available from the corresponding author upon reasonable request.

## Abbreviations

FL: Foreign Language
PP: Positive Psychology
EFL: English as a Foreign Language
CFA: Confirmatory Factor Analysis
SEM: Structural Equation Modelling
CR: Composite Reliability
AVE: Average Variance Extracted
MSV: Maximum Shared Variance
SLA: Second Language Acquisition
POE: Perseverance of Effort
COI: Consistency of Interest
EAP: English for Academic Purposes
CI: Confidence Intervals
TES: Teacher Emotional Support
